# Chitosan-Based Drug Delivery Systems for Targeted Chemotherapy in Colorectal Cancer: A Scoping Review

**DOI:** 10.3390/md23120467

**Published:** 2025-12-06

**Authors:** Urszula Piotrowska, Joanna Szatko, Aleksandra Nowakowska, Emilia Klimaszewska, Marta Ogorzałek, Marcin Sobczak

**Affiliations:** 1 Department of Pharmaceutical Chemistry and Biomaterials, Faculty of Pharmacy, Medical University of Warsaw, 1 Banacha Str., 02-097 Warsaw, Poland; 2 Department of Basic Medical Sciences, Faculty of Medical Sciences and Health Sciences, Casimir Pulaski University of Radom, 27 Chrobrego Str., 26-600 Radom, Poland; 3 Students Scientific Group BIOMAT, Faculty of Pharmacy, Medical University of Warsaw, Banacha 1 Str., 02-091 Warsaw, Poland; 4 Department of Cosmetology, Faculty of Medical Sciences and Health Sciences, Casimir Pulaski University of Radom, 27 Chrobrego Str., 26-600 Radom, Poland

**Keywords:** colorectal cancer, chitosan, targeted drug delivery, ligand modification, nanoparticles

## Abstract

Chitosan (CS) has emerged as a versatile biopolymer for designing drug delivery systems (DDS) in colorectal cancer (CRC) therapy due to its biocompatibility, mucoadhesive properties, and ability to be surface-functionalized. This scoping review systematically analyzed current experimental studies on CS-based DDS for CRC, comparing non-targeted formulations with ligand-modified systems to identify advances in targeting efficiency, drug release behavior, and biological outcomes. Among the twenty-five initially identified studies, divided into two categories, non-targeted CS-based DDSs and ligand-modified CS-DDSs, five fulfilled the inclusion criteria for ligand-functionalized systems. These incorporated targeting moieties, such as folic acid (FA), hyaluronic acid (HA), and galactose (Gal), to achieve receptor-mediated uptake via FRα, CD44, and ASGP receptors, respectively. Ligand modification consistently enhanced cellular uptake, reduced IC_50_ values, and improved tumor-selective cytotoxicity compared to non-targeted systems. However, in vivo validation remains scarce, with only one study confirming tumor accumulation in xenograft models. Moreover, no clinical trials currently assess CS-based nanocarriers for the treatment of CRC. Overall, CS represents a promising modular platform for targeted nanomedicine, but translational progress requires bridging preclinical success with comprehensive in vivo and clinical evaluation.

## 1. Introduction

Cancer is one of the leading causes of illness and death worldwide. Approximately 10 million new cases are estimated each year. Standard cancer treatment methods include chemotherapy, administered as neoadjuvant or adjuvant therapy, and radiotherapy, particularly when surgical removal of the tumor is impossible. However, chemotherapy and radiotherapy regimens are often ineffective due to their lack of specificity for cancer cells. The lack of specificity of conventional chemotherapeutic agents leads to the destruction of not only cancer cells but also healthy cells, consequently causing serious side effects. For this reason, new drug delivery systems (DDSs) for anticancer drugs are being intensively developed [1,2,3].

Colorectal cancer (CRC) is one of the most common malignancies. In its early stages, the disease causes no symptoms and is most often detected during screening tests, such as colonoscopy. The vast majority (approximately 90%) of CRC cases are adenocarcinomas of the large intestine. Chemotherapy and modern targeted molecular therapies are used in both the neoadjuvant and adjuvant settings for CRC treatment. Neoadjuvant chemotherapy aims to reduce tumor burden, facilitating surgical resection and enhancing the likelihood of complete disease remission [4]. Adjuvant chemotherapy, in turn, is intended to improve patient survival by targeting and eliminating residual cancer cells that may remain after surgery [5].

Treatment for CRC includes chemotherapy (e.g., 5-fluorouracil (5-FU), oxaliplatin (Oxa)) and targeted therapy (e.g., cetuximab (CTX), bevacizumab (BVC), encorafenib (ENC)), as well as immunotherapy (e.g., pembrolizumab (PEM), nivolumab (NIVO)) [6]. The choice of drug depends on the stage of the disease, tumor characteristics (e.g., genetic mutations), and the patient’s treatment history. Combination therapies, such as dual therapy with trifluridine/tipiracil (FTD/TPI), are also used to inhibit tumor growth. In recent years, intensive research has been conducted on new polymeric DDSs containing the previously mentioned active substances [7,8].

Given the limitations of conventional therapies, particularly their lack of specificity and associated side effects, increasing attention has been devoted to the development of advanced DDSs that can enhance treatment outcomes.

One way to improve the anticancer effects of drugs is to develop carriers that enable the delivery of effective drug concentrations to diseased sites without affecting normal cells. Examples of nanocarriers include chitosan (CS) nanoparticles (NPs) [9,10].

**Figure 1:**
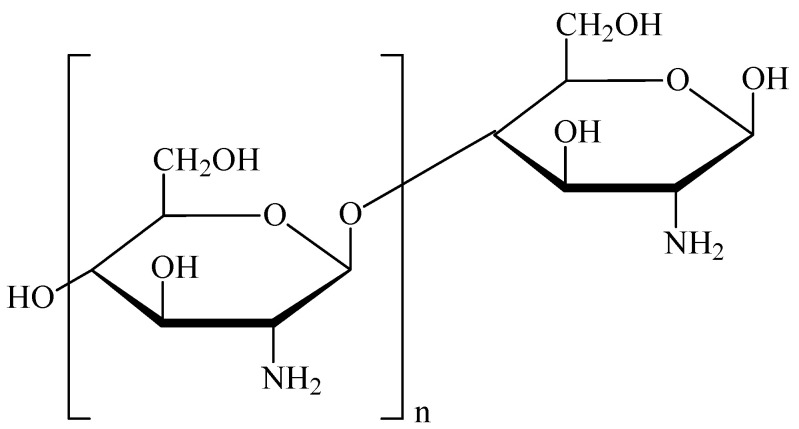
Chemical structure of chitosan.

CS is a non-toxic, biodegradable polymer characterized by high biocompatibility, low immunogenicity, and mucoadhesive as well as absorption-enhancing properties. Its chemical structure is shown in Figure 1.

Although CS has numerous valuable advantages, it also has several limitations that affect its use in the delivery of anticancer drugs. These include low thermal stability [11,12] and ductility [13,14], poor solubility of CS above pH 6.5 [15,16], high hydrophilicity and swelling properties, which may lead to premature drug release and reduced structural stability. Moreover, CS exhibits considerable batch-to-batch variability due to differences in source material, degree of deacetylation, and purity, making reproducibility challenging. Its solubility and viscosity are also sensitive to pH and temperature, respectively, which can further impact formulation stability and processing [17,18,19]. Limitations in sterilization methods also present challenges. Yang et al. [20] demonstrated that steam sterilization significantly darkens CS powder, gamma irradiation causes strong depolymerization above 10 kGy, and ethylene oxide induces only minor changes in crystallinity and structure.

Despite the above limitations, many CS-based DDSs in the form of NPs, hydrogels, and polymeric hydrogel membranes have been developed for oral, ocular, nasal, pulmonary, buccal, periodontal, vaginal, dermal, and transdermal applications, as well as for wound healing, and for vaccine and gene delivery. Among these types of CS-based DDSs, NPs are the most promising formulations for pharmaceutical applications [21,22]. To date, CS-based DDSs have been confirmed to have excellent anticancer properties [23]. Among other things, they are effective against oral cancer, breast cancer, prostate cancer, glioblastoma multiforme, liver cancer, and colon cancer, while also demonstrating satisfactory biocompatibility with typically developing cells and/or tissues. The resulting CS-based DDSs were evaluated for controlled release kinetics and system activation using pH changes [24,25,26,27,28,29,30,31,32,33,34,35,36,37].

Importantly, these systems are also being developed as targeted therapies employing a range of common ligands, such as folic acid (FA), hyaluronic acid (HA), galactose (Gal), and antisense oligonucleotides, which enable receptor-specific interactions with the folate receptor (FR), CD44, and the asialoglycoprotein receptor (ASGPR).

This scoping review aims to explore recent literature on the pharmacological potential of CS-based DDSs for targeted chemotherapy in CRC.

## 2. Review Results

**Figure 2:**
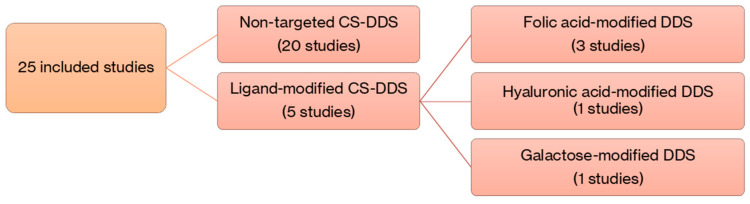
Flowchart showing the classification of the 25 included studies into non-targeted and ligand-modified CS-based DDSs.

This scoping review identified and analyzed a total of 25 original research articles that met the inclusion criteria. These studies were grouped into two categories based on the design of the DDS: (1) CS-based DDS without active targeting ligands, and (2) ligand-modified CS-based DDS for selective drug delivery to CRC cells. This classification is illustrated in Figure 2.

### 2.1. Chitosan-Based DDS

The reviewed literature underscores the versatility of CS as a platform for CRC drug delivery. A total of 20 studies were included in this group, focusing on CS-based delivery systems such as NPs, microparticles (MPs), micelles, and nanogels loaded with various chemotherapeutic and bioactive agents. These included camptothecin (CPT), α-mangostin, irinotecan (CPT-11), curcumin (CUR), imatinib mesylate (IMT), and others. These systems employed diverse formulation strategies to enhance drug stability, enable colon-targeted delivery, and achieve sustained or stimuli-responsive release. Approaches included pH-sensitive coatings, enzyme-triggered mechanisms, magnetic targeting, and polymeric blends.

Most formulations exhibited controlled or pH-responsive release profiles. In vitro assays consistently demonstrated enhanced cytotoxicity of CS-encapsulated drugs compared to their free forms, and several in vivo studies reported superior tumor suppression, reduced systemic toxicity, and prolonged survival in CRC models. The detailed characteristics of these systems are summarized in Table 1.

#### 2.1.1. Routes of Administration and Physiological Barriers

Non-targeted CS-based DDS developed for CRC therapy utilize two principal administration routes: oral colon-targeted delivery and parenteral (intravenous) delivery. Each route encounters distinct physiological barriers. Oral systems must overcome gastric acidity, digestive enzymes, thick intestinal mucus, and first-pass metabolism combining pH-dependent matrices with mucoadhesive or enzyme-responsive components [58,59]. Intravenous formulations, in contrast, face rapid opsonization and clearance by the reticuloendothelial system (RES), restricted vascular permeability, and the acidic, enzyme-rich tumor microenvironment (TME), which may hinder diffusion or destabilize carriers prematurely [60,61,62]. Effective CRC targeting therefore requires DDS able to withstand early degradation while enabling controlled drug release within colonic or tumoral compartments which are characterized by excessive ROS levels, distinct enzyme activity, and a higher pH compared to the upper gastrointestinal tract (GIT) [63].

#### Oral CRC-Targeted Delivery

A substantial proportion of the included non-targeted systems were designed for oral, colon-targeted delivery. Across eight studies, a total of 15 distinct pH-responsive or colon-directed formulations were identified, incorporating polysaccharide matrices, CS derivatives, enteric coatings, and micro-/macroparticle platforms. These systems included CPT-loaded mPEG-CS-OA micelles [38], CS/Alg and TCS/Alg NPs with GP or L100 coatings [39], *Yarrow*-extract MPs/NPs [41], CUR-loaded CS/NaCMC-PLGA hybrids [45], SMV CS ES100 MPs [46], CS-NPs embedded in RS/P MPs [50], ALG-CS MPs/macroparticles [53] and CL-NBSCS systems [57].

Most of these formulations exhibited minimal drug release under gastric conditions and preferential release at colonic pH. For instance, SMV-CS-ES100 MPs prevented significant release until exposure to pH 7.4, achieving complete drug release within 24 h and confirmed colonic deposition via radiographic imaging [46]. Similarly, 5-FU CS-NPs embedded in RS/P MPs prolonged retention in the cecum and colon in vivo while reducing systemic exposure [50]. GP-crosslinked and L100-coated CS/Alg NPs maintained up to 15% release at pH 1.2 and reached 80% release at pH 7.4 within 8 h, demonstrating effective protection of α-mangostin during transit through the upper GIT [39].

Structural modifications of CS further amplified therapeutic efficacy. PEG-OA-modified CS micelles enhanced CPT pharmacokinetics and reduced tumor volume by 50% in murine CRC models [38]. Thiolated CS matrices promoted strong mucoadhesion and resulted in enhanced cytotoxicity compared to free etoposide (ETP) in HCT116 cells [49].

Multiple particulate formulations (both micro- and macroparticles) enabled precise colonic targeting, confirmed by radiographic imaging and biodistribution analyses. These systems ensured controlled systemic exposure and minimized accumulation in non-target tissues [46,50,53].

Collectively, these findings indicate that dual-layer protection, combining an enteric polymer with a CS matrix, provides substantially greater stability and colon-targeting precision than CS-NPs alone, which otherwise exhibit faster release kinetics and measurable systemic absorption.

#### Intravenous Delivery and Systemic Barriers

A total of 13 CS-based DDS across 12 studies were administered intravenously, primarily to enhance systemic circulation time, improve tumor accumulation through the enhanced permeability and retention (EPR) effect, or integrate external or physicochemical triggers to achieve localized drug release within the TME. These systems included polymeric NPs such as IMT-loaded CS-NPs [42] and BEV-loaded CS NPs [47], hybrid CS-based nanocomposites, including CS-polyglutamic acid SN-38 systems co-administered with *Bifidobacterium bifidum* [43], surface-modified CS carriers [44,47], dual-ligand system (ETP-CS-LF-MLT NPs) [49], magnetic polyelectrolyte complexes (MPECs) for magnetically guided targeting [48], metal-CS hybrids [51], CS-stabilized nanostructures, such as CTB-loaded PS-CS-NPs [52] and EV-β-CD-HA-CS-Au nanoclusters (NCs) [56], enzyme- or pH/ultrasound-responsive nanocarriers [54] and charge-reversal Oxa-R-NGs [55].

Following intravenous injection, CS-based DDS must overcome several systemic barriers, including rapid opsonization, RES clearance, vascular endothelial permeability, and the acidic, enzyme-rich TME. Many included systems sought to address these limitations through PEGylation, lipid/CS hybridization, magnetic components, charge-reversal coatings, or enzyme-responsive linkages.

The integration of magnetic elements facilitated both targeted delivery and imaging capabilities. CPT-11-loaded MPECs and SN-38-loaded CS/polyglutamic acid (PGA) nanocomposites demonstrated enhanced tumor localization under magnetic guidance, resulting in higher tumor inhibition with minimal off-target toxicity [40,48].

Advanced nanogels incorporating charge-reversal properties and ultrasound responsiveness achieved 77% oxaliplatin (Oxa) release under tumor-mimicking pH conditions with low-intensity ultrasound, yielding the most pronounced tumor suppression in CT26 xenografts without systemic toxicity [55].

Metal-CS hybrid nanostructures, such as CS-Ag nanourchins (NUs) and CS-Au NCs, further enhanced intracellular uptake through redox-sensitive interactions, improving apoptotic activity while maintaining acceptable biocompatibility profiles [51,56]. BEV-CS-NPs prolonged the half-life of the drug and increased colonic tumor retention, with reduced liver distribution and minimal systemic toxicity in in vivo studies [47].

Safety assessments generally confirmed the high biocompatibility of unmodified CS carriers. However, some hybrid DDSs, such as EV-β-CD-HA-CS-AuNCs, exhibited marginal additive toxicity [56].

Overall, intravenous CS-based DDS demonstrated improved tumor targeting, controlled release within the TME, and enhanced therapeutic efficacy compared with free drugs. However, translation remains limited by the scarcity of immunocompetent animal models, minimal pharmacokinetic and immunogenicity characterization, and high variability in CS composition across studies.

#### 2.1.2. Limitations, Evidence Quality, and Translational Barriers in Non-Targeted Chitosan-Based Drug Delivery Systems

Despite promising efficacy, the overall level of biological evidence supporting non-targeted CS-based DDS remains limited. A significant methodological limitation across studies was the predominant reliance on two-dimensional (2D) monolayer cell cultures (e.g., HT-29, HCT116), with limited exploration of three-dimensional (3D) models or metastatic CRC cell lines. Moreover, the pharmacokinetic and immunogenicity profiles remain underreported for most systems. Only a minority included in vivo validation, and the depth of pharmacokinetic or immunogenicity analyses was generally insufficient to support translational development. For example, capecitabine (CTB)-loaded CS NPs resulted in approximately 72% tumor reduction within 21 days in a DMH-induced CRC mouse model, while concurrently downregulating pro-angiogenic and inflammatory mediators [52], yet such promising outcomes remain isolated and insufficiently validated across independent studies.

Another concern relates to the credibility and robustness of reported efficacy data. In microbiome-assisted delivery, SN-38 CS-based systems co-administered with *Bifidobacterium bifidum*, achieved approximately 80% tumor suppression, doubling the efficacy observed without probiotic co-treatment [43]. However, such high inhibition rates were often reported from short-duration studies with unclear or unreported sample sizes, and lacking key methodological details. Consequently, these outcomes should be interpreted with caution until verified in larger, statistically powered in vivo experiments. In many cases, studies lacked appropriate comparator groups, such as standard chemotherapeutic regimens, making it difficult to determine the true clinical relevance of the observed improvements.

Furthermore, most in vivo studies relied on xenograft models in immunocompromised mice, which do not replicate the human immune response, stromal architecture, or cytokine profile influencing NPs clearance and tumor penetration. As a result, tumor accumulation and antitumor efficacy may be overestimated compared with immunocompetent hosts. Models lacking intact immune surveillance also fail to predict potential immunogenicity or long-term biosafety of CS-based DDS.

A substantial barrier is the heterogeneity of CS materials used across formulations. Formulations varied widely in molecular weight, degree of deacetylation, viscosity grade, type of cross-linking agents, density, and purification source, critically affect solubility, charge distribution, mucoadhesion, and cellular uptake. Due to inconsistent reporting, direct comparisons between formulations are limited and broader generalization is not possible.

Although non-targeted CS systems demonstrated several CRC-relevant functionalities, such as pH-responsive release, magnetic guidance, mucoadhesion, and microbiome-assisted delivery, the absence of standardized experimental methodologies and heterogeneous in vivo endpoints further undermine comparability and reproducibility.

Across diverse drug classes, including fluoropyrimidines, kinase inhibitors, topoisomerase inhibitors, and phytochemicals, CS-based carriers consistently improved antitumor activity through better drug retention, controlled release, and preferential tumor targeting in preclinical screening, but current evidence is insufficient to support translation. Their favorable safety profiles further support CS as a promising core material for next-generation CRC delivery platforms. Reported improvements in tumor inhibition or IC_50_ reduction must therefore be regarded as preliminary signals, not definitive therapeutic advantages.

Nonetheless, clinical translation is currently constrained by methodological inconsistencies, limited long-term toxicity data, and insufficient pharmacokinetic characterization. Addressing these gaps through standardized evaluation protocols and more rigorous in vivo testing is imperative to facilitate the advancement of CS-based systems toward clinical application.

### 2.2. Ligand-Modified CS-Based DDS

**Figure 3:**
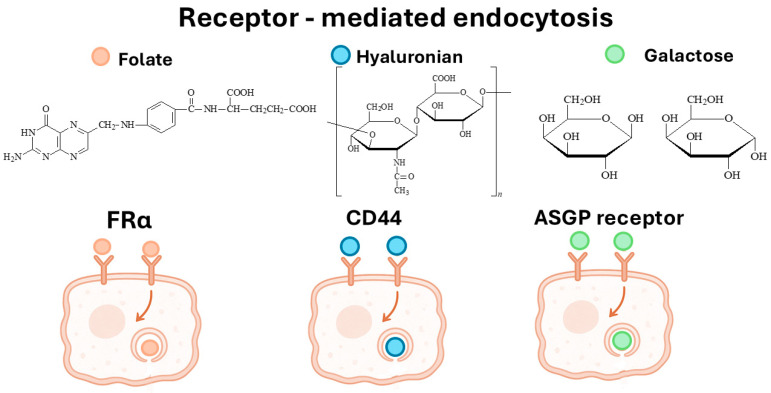
Receptor-mediated endocytosis in colorectal cancer cells via FRα, CD44, and ASGP receptors, showing ligand binding preferences and internalization pathways.

To improve the specificity of CS-DDSs, various ligands have been incorporated to enable receptor-mediated targeting of CRC cells. Among the most commonly used are FA, HA, Gal, and antisense oligonucleotides, which enable receptor-specific interactions with the FR, CD44, and the ASGP receptor (Figure 3). These ligand-functionalized systems consistently demonstrated enhanced cellular uptake, improved cytotoxicity, and stronger tumor localization compared to non-targeted CS carriers. Accordingly, IC_50_ values were significantly reduced, and therapeutic efficacy was improved both in vitro and, in one instance, in vivo.

Although nine studies were initially identified, two review articles [64,65] were excluded as non-original research. Among the remaining seven experimental papers, two were removed during full-text screening due to the absence of active ligand-receptor targeting. Both employed passive targeting mechanisms, such as EPR or physicochemical selectivity, rather than specific molecular interactions. The gold nanocomposite system developed by Tan et al. [56] was reclassified under non-targeted DDSs owing to its passive physicochemical targeting. In contrast, the mesoporous silica NPs described by AbouAitah et al. [66] relied solely on EPR-driven tumor accumulation and were excluded from the comparative analysis.

The five ligand-modified CS-based DDS that met the inclusion criteria and demonstrated receptor-mediated active targeting are summarized in Table 2, along with their key design characteristics, release behavior, and biological outcomes.

#### 2.2.1. Folic Acid

FA is a targeting ligand composed of pteroic acid and glutamic acid connected via an amide bond [72]. It is one of the most extensively explored ligands in DDSs, providing an efficient strategy for the treatment and imaging of various cancers and inflammatory diseases. Due to its small molecular weight and high affinity for the FR, which is overexpressed in malignant cells but minimally expressed in healthy tissues, FA enables selective delivery of therapeutic and diagnostic agents to pathological cells without affecting normal ones [73].

Hamed et al. [74] reported the formation of metal complexes with the folate anions. Their results indicated that two FA complexes were formed in a 1:2 molar ratio (metal:FA), where FA acted as a bidentate ligand through both carboxyl groups. Polarized light studies confirmed that the FA complexes possessed a symmetric geometry. The authors concluded that transition metal-FA complexes are more suitable as therapeutic agents than free FA, owing to their higher absorption efficiency in biological systems.

Kola et al. [75] used NMR and spectroscopic studies to elucidate the interaction of FA with Cu(II) and other metal ions at varying concentrations. They observed that Cu^2+^ primarily coordinated with the pteridine ring (PTE) of FA, with minimal involvement of the glutamic acid moiety. This interaction was concentration-dependent: at lower FA concentrations, Cu^2+^ effectively bound to the PTE, whereas at higher concentrations, intermolecular interactions between FA molecules hindered copper coordination. Pronounced paramagnetic effects were detected on the PTE and p-aminobenzoic acid regions, with negligible influence on Glu signals. These findings provide new structural insight into FA-Cu^2+^ complexation, advancing the understanding of folate coordination behavior as a ligand in solution.

McMullon et al. [76] synthesized folate conjugates with stable lanthanide complexes, highlighting their potential application as luminescent and MRI-active probes for targeting FR-expressing cells.

Ragab et al. [77] developed binuclear Mn(II) complexes incorporating FA and co-ligands (Bpy/Phen), which exhibited significant cytotoxic activity against FR-positive cancer cell lines, particularly HCT116 colorectal cancer cells. The study also investigated the anticancer mechanisms, including wound-healing inhibition, cell cycle arrest, regulation of apoptotic proteins, and morphological alterations. These results lay the groundwork for future research exploring the therapeutic and diagnostic potential of FA-based metal chelates as targeting ligands.

Overall, FA conjugation significantly improved selective drug internalization into FRα-overexpressing CRC cells, reduced off-target toxicity, and enhanced cytotoxicity for agents such as 5-FU, IMT, and polyphenols.

Consistent with these findings, FA-conjugated CS NPs demonstrated superior internalization in FR-overexpressing colorectal cancer cells and significantly enhanced the cytotoxicity of 5-FU and RSV/FER combinations compared with non-targeted CS carriers [67,69].

Similarly, mesoporous silica nanoparticles (MSNPs) functionalized with FA exhibited strong FR-mediated uptake and selective cytotoxicity toward HT-29 cells, confirming the effectiveness of FA as a targeting ligand for site-specific CRC therapy [68].

**Figure 4:**
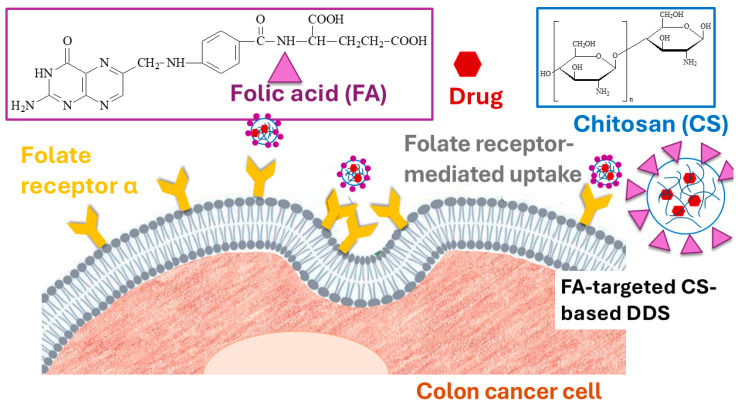
Schematic representation of FRα-mediated internalization of folate-targeted CS-based DDSs. FA on the nanocarrier surface binds to overexpressed folate receptors on CRC cells, triggering receptor-mediated endocytosis and facilitating intracellular delivery of the encapsulated drug.

These findings collectively confirm the critical role of FR-mediated targeting in improving the selectivity and potency of CS-based DDS against CRC. To visualize the uptake behavior of folate-targeted CS-based DDSs, Figure 4 presents the FRα-mediated endocytic pathway responsible for their selective internalization into CRC cells.

However, the therapeutic benefit of FA-based targeting strongly depends on the heterogeneous expression of folate receptors across CRC subtypes. Moreover, surface binding limits deeper tumor penetration, a phenomenon not addressed in current studies [78].

#### 2.2.2. Hyaluronic Acid

HA is a naturally occurring linear polysaccharide belonging to the glycosaminoglycan family. It is a significant component of the ECM in connective, epithelial, and neural tissues of the human body, with over half of its total content found in the skin. Its chemical structure consists of repeating disaccharide units of D-glucuronic acid and N-acetyl-D-glucosamine linked by β-1,4 and β-1,3 glycosidic bonds [79,80].

HA exhibits distinctive physicochemical properties, including high hygroscopicity, viscoelasticity, biocompatibility, and non-immunogenicity. The biological role of HA depends strongly on its molecular weight: high-molecular-weight HA (HMW-HA, >1000 kDa) maintains hydration, lubrication, and tissue integrity, exerting anti-inflammatory and immunosuppressive effects; conversely, low-molecular-weight HA (LMW-HA) fragments act as signaling molecules that activate inflammatory responses, promote angiogenesis, and facilitate cell migration during tissue repair [81,82].

In pharmaceutical applications, HA has been widely utilized as a carrier material due to its excellent biocompatibility, biodegradability, non-toxicity, and specific interactions with cell surface receptors such as CD44. These characteristics make HA an attractive ligand and matrix component in the design of targeted DDSs [83,84,85].

Consistent with its biological affinity for the CD44 receptor, HA-based CS or PLGA nanocarriers exploited CD44 overexpression, a major driver of CRC stemness and metastasis, to achieve enhanced tumor uptake, robust anti-proliferative activity, and reduced cell migration compared with free apigenin or uncoated NPs [70]. In vivo imaging confirmed efficient tumor accumulation of HA-coated NPs with minimal off-target distribution, validating CD44-mediated active targeting as a powerful strategy for CRC therapy.

Nonetheless, CD44 expression varies widely between CRC stages [86,87]. This heterogeneity may limit uniform nanoparticle uptake across entire tumors. Additionally, high molecular weight HA may exhibit slower internalization rates, whereas low molecular weight HA may induce pro-inflammatory signaling—factors not systematically assessed in current studies [88].

#### 2.2.3. Galactose

Gal is a monosaccharide found in lactose, glycoproteins, and glycolipids, and occurs in specific polysaccharides. In the human body, it can be metabolized to glucose. D-Gal has been extensively investigated as a targeting ligand in DDSs rather than as a primary carrier. Its main function is its specific affinity for ASGPR, which are abundantly expressed on hepatocytes and also present on specific cancer cells and macrophages. The covalent attachment of Gal to drug molecules or to NPs/liposomes enhances receptor-mediated uptake, thereby improving targeted delivery efficiency [89,90,91].

D’Souza et al. [92] provided a comprehensive overview of ligand-receptor strategies for hepatocyte-targeted delivery via ASGPR. They emphasized that sugar isomerism, Gal density and branching, spatial geometry, and glycosidic linkage patterns are key factors influencing receptor binding. Computational docking studies further support the design of synthetic multivalent Gal/GalNAc ligands tailored for improved receptor recognition and binding strength.

Ma et al. [93] developed a Gal-based fluorescent probe (Gal-MPA) to assess its binding affinity toward cancer cells. The probe exhibited markedly higher affinity for cancer cell lines (HepG2, MCF-7, A549) than for normal liver cells (L02), confirming the potential of Gal as a broad, tumor-targeting ligand for imaging and therapy.

Carbohydrate-mediated targeting was further demonstrated in galactosylated CS-functionalized MSNPs, which achieved selective cytotoxicity toward HT-29 colon cancer cells via ASGPR recognition, while exhibiting reduced toxicity toward normal fibroblasts [71]. These results support the application of Gal as an effective targeting ligand for site-specific CRC drug delivery.

However, ASGPR expression in CRC is heterogeneous within CRC lesions [94]. Therefore, while Gal enhances uptake in some CRC models, its broader applicability may be limited. Future studies should stratify CRC models by ASGPR status to clarify its true therapeutic value.

#### 2.2.4. Summary and Challenges of Ligand-Modified CS-Based DDS

To provide a comparative overview of the main targeting strategies, Table 3 summarizes the ligands applied in CS-based DDS for CRC, their corresponding receptors, expression profiles, mechanisms of receptor-mediated uptake, and biological effects reported in the included studies [67,68,69,70,71]. The three principal ligands identified, FA, HA, and Gal, enable receptor-specific targeting of CRC-associated biomarkers, thereby improving drug localization and efficacy.

Overall, ligand modification of CS-based DDS consistently enhanced tumor-selective uptake, reduced IC_50_ values, and induced stronger apoptotic responses compared to non-targeted systems and free drugs. These findings underscore the versatility of CS as a modular platform for ligand conjugation and surface functionalization, enabling the exploitation of CRC-specific molecular signatures.

FA-conjugated CS NPs demonstrated superior internalization in FR-overexpressing CRC cells and significantly improved cytotoxicity of 5-FU and RSV/FER combinations compared with non-targeted systems [67,69]. Similarly, FA-functionalized CS-coated Zn–MOF nanocomposites exhibited pronounced FR-mediated uptake and vigorous proapoptotic and autophagic activity in HCT116 cells [68]. HA-based systems effectively targeted CD44, overexpressing CRC phenotypes, key drivers of stemness and metastasis, showing enhanced tumor accumulation, reduced migration, and selective cytotoxicity compared with free apigenin [70]. Carbohydrate-mediated targeting was also confirmed in galactosylated CS-functionalized MSNPs, which achieved selective cytotoxicity toward HT-29 cells through ASGPR recognition while sparing non-cancerous fibroblasts [71].

Across all ligand types, receptor heterogeneity, binding-site barrier effects, and variable ligand density present unresolved limitations that may diminish targeting consistency in vivo. None of the included studies systematically optimized ligand density or assessed saturation kinetics, which are crucial for translational performance.

Despite these promising in vitro results, the translational maturity of ligand-modified CS-based DDS remains limited. Most studies were restricted to 2D monolayer models, with only one demonstrating in vivo targeting efficiency. Data on pharmacokinetics, biodistribution, and long-term biosafety are still lacking. Bridging the gap between enhanced molecular targeting and clinical relevance requires systematic evaluation in 3D tumor organoids and xenograft models, supported by in vivo validation to confirm whether receptor-mediated selectivity observed in vitro can translate into meaningful therapeutic benefit.

### 2.3. Comparative Analysis: Non-Targeted vs. Ligand-Modified CS-Based DDS

A comparative evaluation of the included studies revealed distinct performance differences between non-targeted and ligand-modified CS-based DDS in terms of cellular uptake, cytotoxicity, TME penetration, toxicity profile, and targeting efficiency.

These differences arise not only from the presence of a ligand but also from receptor density, ligand-receptor binding affinity, NPs surface charge, and steric accessibility of the targeting moiety, factors that were rarely controlled or compared across studies.

#### 2.3.1. Cellular Uptake and Cytotoxicity

Ligand conjugation significantly enhanced receptor-mediated internalization efficiency compared to passive uptake mechanisms in non-targeted systems. FA- and HA-modified CS-DDS showed up to a 1.5–3-fold increase in cellular uptake in FRα^+^ and CD44^+^ colorectal cancer cells, respectively. Correspondingly, IC_50_ values were reduced by 40–70% relative to non-functionalized CS carriers, indicating enhanced intracellular drug accumulation and potency. In contrast, non-targeted CS NPs primarily relied on electrostatic interactions or EPR effects, yielding slower uptake kinetics and higher IC_50_ values.

#### 2.3.2. Tumor Microenvironment Penetration

Ligand-modified DDSs exhibited superior penetration in TME-mimicking models, attributed to receptor-mediated internalization, enhanced mucoadhesion, and improved stability under acidic pH (6.5–6.8). FA and HA functionalization provided additional protection against premature drug release in neutral physiological conditions (pH 7.4) while enabling responsive release in mildly acidic or enzymatically active environments, consistent with CRC tumor pH gradients.

#### 2.3.3. Toxicity Profile

Ligand-functionalized CS-based DDS generally exhibited lower systemic and off-target toxicity than free drugs and non-targeted systems. Enhanced tumor specificity reduced undesired cytotoxicity in normal fibroblast and epithelial cells. Moreover, the biocompatible nature of CS and its derivatives contributed to reduced gastrointestinal irritation and improved tolerability, as reported in both in vitro and limited in vivo assessments.

#### 2.3.4. Summary

A comparative synthesis of non-targeted (Table 1) and ligand-modified (Table 2) CS-based DDS is presented in Table 4.

In summary, ligand modification significantly outperformed non-targeted CS-DDS in terms of cellular uptake, cytotoxicity, and tumor-specific accumulation, while maintaining excellent biocompatibility. Among active targeting ligands, FA-based systems exhibited the most substantial receptor-mediated uptake and cytotoxic response, followed by HA- and Gal-functionalized formulations. HA-CD44 targeting proved particularly effective against metastatic and stem-like CRC phenotypes, whereas Gal-ASGPR interactions enhanced selectivity and minimized fibroblast toxicity.

Despite these promising findings, the in vivo efficacy of active targeting remains insufficiently validated. Only one study demonstrated successful tumor accumulation in xenograft models, while the remaining investigations were restricted to 2D monolayer or co-culture systems. The absence of comprehensive in vivo data, pharmacokinetic profiling, and long-term biosafety evaluation continues to hinder clinical translation. Future comparative studies integrating 3D tumor organoids and relevant animal models are required to bridge this preclinical gap and confirm whether the enhanced molecular specificity of ligand-modified CS-DDS translates into meaningful therapeutic benefit.

## 3. Clinical Perspective

Only a few clinical trials (ClinicalTrials.gov, accessed 11 November 2025) involving CS in cancer therapy have been registered to date, and none directly evaluate CS-based nanocarriers for CRC. The identified studies include: prostate cancer (NCT03712371), cancer-related breakthrough pain (NCT02591017), post-surgical applications in breast cancer (NCT02967146), lung cancer prognosis (NCT04218188), and sellar reconstruction in skull base surgery (NCT03280849). Additional studies investigated the use of CS in laser-assisted immunotherapy for advanced breast cancer (NCT03202446), intratumoral injection following thermal ablation in advanced solid tumors (NCT03993678), and topical or local treatments for oral lesions, including oral lichen planus (NCT07114016, NCT06135259) and oral squamous cell carcinoma (NCT05893888). Collectively, these trials primarily focus on supportive, surgical, or topical therapeutic applications rather than systemic anticancer drug delivery, underscoring the lack of translational or clinical evaluation of CS-based DDS in oncology.

## 4. Materials and Methods

This scoping review was conducted in accordance with the PRISMA-ScR (Preferred Reporting Items for Systematic Reviews and Meta-Analyses extension for Scoping Reviews) guidelines [95]. The objective was to systematically map the research landscape concerning CS-based DDS for chemotherapy in CRC, focusing on both general DDS applications and ligand-mediated targeted systems.

### 4.1. Search Strategy

Two complementary literature search strategies were employed to ensure comprehensive coverage of relevant studies. The first strategy focused on identifying research related to general CS-based DDSs for CRC treatment, without emphasizing active targeting. The second strategy targeted studies involving CS-based DDSs modified with active targeting ligands such as folic acid, hyaluronic acid, peptides, antibodies, or other tumor-specific molecules.

A two-stage approach was applied. First, a broad MeSH-based query retrieved all potentially relevant articles (Section 1 search string), ensuring sensitivity and a full landscape of available evidence. Second, a more specific Title/Abstract keyword search focused on ligand-based active targeting strategies (Section 2 search string), was conducted enhancing specificity and yielding the final screening pool.

Both searches were conducted in PubMed and limited to original research articles written in English, published between January 2020 and June 2025. This timeframe was selected to capture the most recent trends in CS-based DDSs for CRC. Reviews, editorials, conference abstracts, and non-CRC studies were excluded.

**Figure 5:**
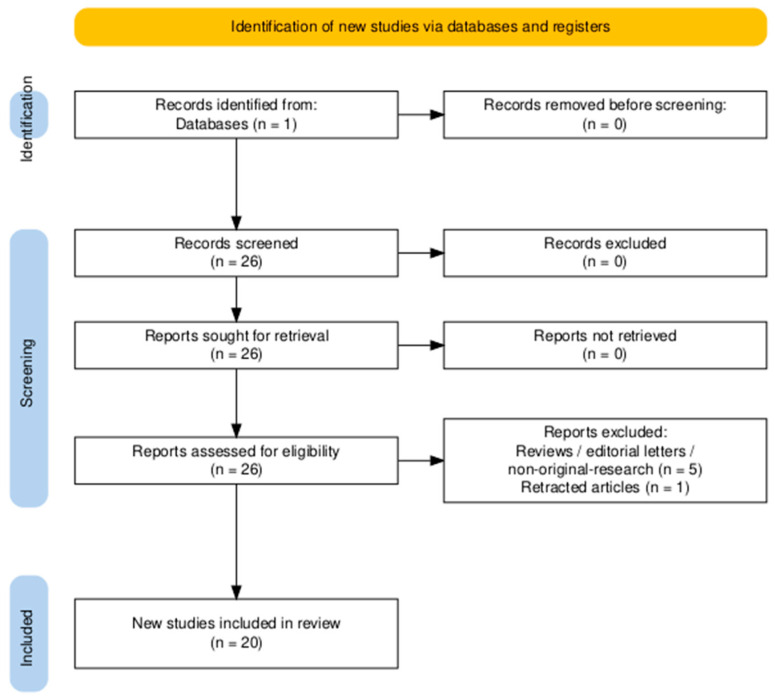
PRISMA 2020 flow diagram detailing the identification, screening and inclusion stages for studies investigating chitosan-based drug delivery systems for colorectal cancer. Diagram generated using the PRISMA2020 R package and the PRISMA Shiny app [96].

**Figure 6:**
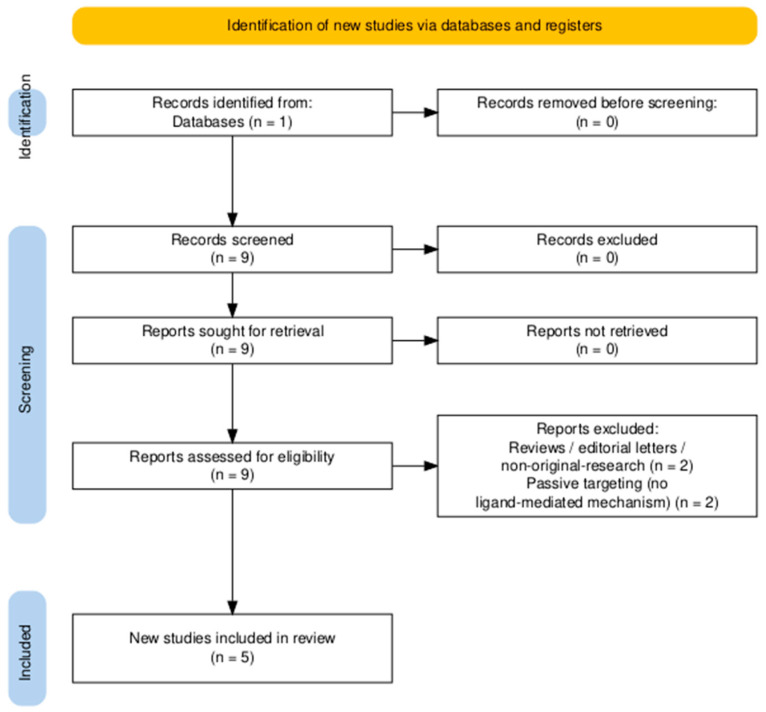
PRISMA 2020 flow diagram illustrating the identification, screening, and inclusion process for ligand-modified chitosan-based drug delivery systems for colorectal cancer. Diagram generated using the PRISMA2020 R package and the PRISMA Shiny app [96].

Full Boolean expressions are available in the Supporting Information (Table S1), with corresponding search trees illustrated in Figure 5 and Figure 6.

### 4.2. Inclusion and Exclusion Criteria

To be included in this review, studies had to meet the following eligibility criteria: (1) they had to report on a CS-based DDS developed for the treatment of CRC, (2) include a chemotherapeutic payload, and (3) present experimental in vitro and/or in vivo evaluation of biological activity.

Studies were excluded if they were not focused on CRC, did not involve CS in the DDS composition, lacked a chemotherapeutic agent, or did not present any biological results. Additionally, review articles, book chapters, and papers written in languages other than English were excluded.

### 4.3. Data Screening and Extraction

After removing duplicates, all identified records were screened in a two-stage process. First, titles and abstracts were reviewed to eliminate irrelevant studies. Then, the full texts of potentially eligible articles were assessed. Four independent reviewers conducted the screening and data extraction, while discrepancies were resolved through discussion with a fifth reviewer.

Extracted data included the type and composition of the DDS, the presence and type of ligands used (if applicable), the chemotherapeutic agent, drug release methodology and outcomes, cytotoxicity results with specific cell lines, animal models used in vivo, and the biological effects observed.

The studies were then categorized into two thematic groups. The first group included CS-based DDS for CRC without necessarily incorporating targeting ligands and is discussed in Section 2.2 and summarized in Table 1. The second group comprised ligand-modified DDS designed for targeted therapy and is presented in Section 2.3, with corresponding data in Table 2.

## 5. Conclusions

Despite remarkable in vitro and preclinical progress, the clinical translation of CS-based DDSs, particularly those employing ligand-mediated targeting, still remains limited. None of the ligand-modified CS-based nanocarriers identified in this review has advanced beyond preclinical testing, underscoring the persistent challenges that impede transition from laboratory to clinical application.

The development of novel CS-based therapeutic systems containing anticancer drugs with effective therapeutic potential is a future challenge for modern oncology. By enhancing drug stability, bioavailability, and tumor selectivity, CS NPs in oncology present a significant opportunity to overcome the shortcomings of standard therapies, enabling early and effective treatment. Further intensification of scientific and development work in this area is expected in the coming years.

## Figures and Tables

**Table 1:** Summary of chitosan-based drug delivery systems for colorectal cancer treatment.

DDS	Drug	Drug Release Study	In Vitro Studies		In Vivo Studies		Ref.
			Cytotoxicity	Cell Line	Animal Model	Effects	
CPT-loaded mPEG-CS-OA micelles	CPT	**-**	Empty micelles were non-toxic; CPT-loaded micelles reduced CPT toxicity (0.025–250 μg/mL; 24–72 h). Reduced metabolic activity and spheroid size in MCTS; enhanced cytotoxicity vs. free CPT.	PBMC Caco-2 HT29 HCT116 (2D and 3D MCTS)	C57BL/6 mice (pharmacokinetics study), NIH(S)II-nu/nu mice (HCT116 xenograft), AOM/DSS-induced CRC.	CPT-micelles delayed tumor growth, reduced volume by c.a. 50%; increased survival vs. free drug; confirmed tumor targeting.	[38]
CS/Alg NPs GP-CS/Alg NPs L100-GP-CS/ALG NPs TCS/Alg NPs GP-TCS/Alg NPs L100-GP-TCS/ALG NPs	α-Mangostin	Free drug showed complete release within 2–3 h. Non-crosslinked CS/ALG and TCS/ALG NPs released α-mangostin fully within 6–7 h and 5–6 h, respectively. At pH 1.2, CS/ALG NPs showed higher diffusion, while TCS/ALG NPs released more drug at pH 6.8–7.4. GP crosslinking slowed diffusion and degradation, resulting in similar sustained release for GP-CS/ALG and GP-TCS/ALG NPs across all pHs. L100 coating further reduced early release (<15% at pH 1.2; 35% at pH 6.8); at pH 7.4, 65% was released by 6 h and 80% by 8 h, indicating colon-targeted delivery.	Dose-dependent cytotoxicity toward HT-29; 12–15% viability at 400–600 µg/mL (24 h); apoptosis-related anti-tumor activity; blank CS/ALG and TCS/ALG NPs (0–600 mg/mL) non-toxic (NHF > 90% viability), α-mangostin-loaded NPs safe up to 300 µg/mL; NHF viability > 80%.	NHF HT-29	-	-	[39]
CPT-11/Fe_3_O_4_/CS-PG A-PECs	CPT-11	pH-dependent release: slow at pH 7.4 (plateau at 48 h), faster at pH 6.5 (complete in 24 h) and pH 5.0 (complete in 12 h), enabling targeted tumor delivery.	blank MPECs non-toxic up to 2400 µg/mL; CPT-11-loaded MPECs are more cytotoxic than free CPT-11 (IC_50_: 25.1 vs. 39.2 µM).	HUVEC HCT116	BALB/c nude mice xenograft (HCT-116)	Real-time NIR imaging revealed strong tumor-localized fluorescence at 24–48 h for ICG/MPECs compared to weak signal for free ICG and non-MPECs indicating effective magnetic targeting with high tumor specificity and no systemic toxicity.	[40]
Ionic gelation NPs (LCS:TPP 5:1); Spray-dried MPs (CS:extract 6:1)CS MPs/NPs	*Yarrow* extract (chlorogenic acid, 1,5- and 3,5-DCQAs)	NPs: pH 2—chlorogenic acid 59.6% (5 min), 1,5-DCQA 20.8% (3 h); >75% phenolics retained after 3 h. pH 7.4—slightly lower release. MPs: chlorogenic acid 56.9% (3 h); DCQAs 9.6–37%; luteolin dihexoside 33%. After simulated GI digestion, 65–97% phenolics remained encapsulated.	IC_50_ values after 48 h of treatment with the extract: 78.6 µg/mL.	DLD-1	-	-	[41]
IMT-PNPs NPs	IMT	IMT: c.a. 100% release at 12 h. IMT-PNPs: 86.45 ± 0.05% cumulative release over 84 h; initial burst (0–2 h) followed by sustained diffusion-controlled release; zero-order kinetics (r^2^ = 0.9985).	Dose-dependent cytotoxicity; placebo CS NPs slightly reduced viability (10–15%) due to intrinsic uptake by tumor cells; IMT-PNPs showed significantly higher cytotoxicity vs. free IMT (*p* < 0.0001 at 20 µg/mL).	CT-26	-	-	[42]
CS-L-PGA-SN38 NPs/*B. biffi*	SN-38	20% release (120 h) without esterase presence and 70% release (120 h) with esterase; esterase-triggered; without apparent premature leakage in normal tissue.	Blank CS-L-PGA NPs (0.125–1.5 mg/mL) and 1.6 × 10^5^ to 1.6 × 10^10^ cfu/mL *B. bifi* had minimal impact on the cellular activities of HT-29 and HUVEC. *B. bifi* metabolites—dose-dependent cytotoxicity on HT-29 cells; IC_50_ = 0.57 µg/mL (free SN-38), 1.02 µg/mL (CS-L-PGA NPs), 1.26 µg/mL CS-L-PGA NPs/*B. bifi*) efficient cellular uptake and endocytosis-driven internalization.	HUVEC HT-29	ICR mice HT-29 tumor xenograft model on BALB/c nude mice (biodistribution study).	Tumor inhibition 80% CS-L-PGA-SN38 NPs/*B. bifi* vs. 40% (CS-L-PGA-SN38 NPs); improved survival and body weight; reduced diarrhea and systemic toxicity; strong tumor localization; apoptosis confirmed by condensed nuclei; no organ damage, biosafety.	[43]
CS-IMT-NPs CS-IMT-TPGS-NPs	IMT	pH 5.5: 91.06 ± 5.31% (CS-IMT-NPs), 96.31 ± 3.19% (CS-IMT-TPGS-NPs); pH 6.8: 71.03 ± 3.89% and 86.06 ± 3.73%; pH 7.4: 79.89 ± 2.10% and 83.06 ± 2.90%. pH-sensitive sustained release behavior (no burst). Korsmeyer-Peppas model best fit (R^2^ = 0.9871/0.9745; n = 0.356–0.390): non-Fickian (anomalous) diffusion; sustained, predictable release under acidic pH.	CS-IMT-TPGS-NPs had a significantly lower IC_50_ value of 6.77 μg/mL, compared to free IMT at 13.77 μg/mL; reduced ROS to 22.85% with CS-IMT-TPGS-NPs in comparison to the free IMT and CS-IMT-NPs; and enhance uptake.	HCT116	DMH-induced CRC model in Wistar rats.	Tumor volume reduced by 76.2% vs. 37.6% (IMT); fewer aberrant crypt foci; enhance cellular uptake; reduced ROS; improved colon-specific targeting and biocompatibility.	[44]
CUR loaded CS/NaCMC-PLGA	CUR	pH-dependent sustained release (pH 4.0: 43%, pH 7.4: 69% after 7 day).	CUR-NPs are less cytotoxic than the free drug, with dose-dependent cytotoxicity.	HCT116	-	-	[45]
SMV-CS-ES100 MPs	SMV	pH-controlled, SMV released from MPs did not exceed 10% until 4 h (pH values 1.2 and 4.5). After 4 h at pH 7.4, SMV release increased dramatically reaching 100% within 24 h.	Induced strong dose-dependent antiproliferative and pro-apoptotic effects. Cell cycle analysis showed accumulation in G_2_/M and pre-G_1_ phases; pre-G_1_ increased from 2.1% (control) to 31.5% with SMV-CS-ES100 MPs, vs. 16.5% for free SMV. Enhanced apoptosis and cell death via mevalonate pathway inhibition and caspase-3 activation.	HCT116	New Zeland male rabbits (iohexol radiography).	Real-time X-ray radiography showed colon-specific targeting with delivery to the colon at 6–9 h (post-dose).	[46]
BEV-CS-NPs	BEV	pH-dependent sustained release. At neutral pH, Higuchi-type diffusion is observed. Korsmeyer-Peppas model best fit; non-Fickian diffusion. BEV-CS-NP showed prolonged release.	IC_50_ = 633.0 ± 23.0 μg/mL (3–48 h exposure) IC_50_ = 336.4 ± 20.8 (7–6 day exposure) due to a probable degradation of CS-based NP.	Colon tumor organoids (HCTO)	*Apc^min/+^* mice on C57BL/6J	Moderately high tumor Kp (4.23) Prolonged colon retention, reduced liver and brain distribution and, reduced systemic toxicity. Pharmacokinetics: V_d_ = 2036 L, Cl = 577 L/h, t_½β_ = 44.2 h.	[47]
CS/PGA-SN-38 PECs CS/PGA-SN-38 MPECs	SN-38	Sustained release in PBS (pH 7.4); initial burst < 12 h (≤20% release); 75% after 72 h with esterase (PGA-SN-38 > 90%), enzyme-responsive, tumor-microenvironment-dependent release.	Blank MPECs (100–2000 μg/mL) showed negligible cytotoxicity in HUVECs and HCT-116 cells after 48 h. MPECs showed dose-dependent cytotoxicity on HCT-116. CS/PGA-SN-38 MPECs’ magnetic targeting ability and accumulation in the tumor cells via endocytosis.	HUVEC, HCT116	HCT-116 colorectal xenograft model on BALB/c nude mice.	Enhanced tumor accumulation under magnetic field; a dose of 2.5 mg/kg equivalent SN-38 in MPECs resulted in up to 82% inhibition of tumor growth by day 22; 30% of improvement of tumor regression. No systemic toxicity.	[48]
ETP-CS-LF-MLT-NPs	ETP	Sustained release over 24 h; pH 5.5: 98.7%, pH 6.8: 70.7%, pH 7.4: 60.7%. Acidic pH enhanced release; no burst effect. The release followed the Korsmeyer–Peppas model (R^2^ = 0.9892, n < 1; Fickian diffusion).	IC_50_ (HCT-116): ETP = 419.8 μg/mL; ETP-CS-NPs = 53.7 μg/mL; ETP-CS-LF-NPs = 99.6 μg/mL; ETP-CS-LF-MLT-NPs = 25.4 μg/mL, 16× higher cytotoxicity vs. free drug; Wound healing assay: >94% closure at 24 h, enhanced migration and proliferation without toxicity; MMP reduction: strong mitochondrial depolarization (MFI = 30.5 vs. 65.8 control), indicating apoptosis induction.	HCT116	Wistar rats (DMH-induced CRC model); Albino Wistar rats (PK and biodistribution; HET-CAM (egg model).	Hemolysis: 0.82% (NPs), 0.73% (ETP): non-hemolytic; no platelet aggregation. HET-CAM: 75.6% inflammation reduction, anti-angiogenic effect. Pharmacokinetics: 25.5× longer MRT vs. ETP, increased C_max_ and AUC. Biodistribution: high in GI tract, strong colon uptake. Efficacy: reduced adenocarcinoma growth, improved histology, no toxicity (50 mg/kg, 14 days).	[49]
CS NPs-loaded RS/P MPs	5-FU	pH-dependent sustained release: CS NPs: 91.1% release at pH 1.2 (120 min); CS NPs-loaded RS/P MPs (up to 420 min: 120 min at pH = 1.2; 240 min at pH = 6.8; up to 60 min at pH = 7.4). Release kinetics: CS NPs: Korsmeyer–Peppas model, non-Fickian diffusion. CS NPs-RS/P MPs: Weibull model (n > 1), complex mechanism.	-	-	Female BALB-c mice.	CS NPs: Rapid GI transit; partial accumulation in blood and kidneys. CS NPs-loaded RS/P MPs: Prolonged retention in cecum/colon; minimal systemic distribution.	[50]
IMT-CS-AgNUs	IMT	At pH = 6.8: IMT released 90% in 6 h and c.a. 100% in 12 h; IMT-CS-NPs released 70% in 6 h and c.a. 100% in 12 h; IMT-CS-AgNUs released c.a. 60% in 6 h and 85% in 12 h.	IC_50_: IMT 1.8 µM, IMT-CS-NPs 0.9 µM, IMT-CS-AgNUs 0.4 µM. Apoptosis (early/late %): IMT 17.5/12.9, NPs 24.7/18.3, AgNUs 35.2/29.8. ROS levels (AU): control 10k, IMT 20k, NPs 30k, AgNUs 40k. MMP loss (%): 47.3, 63.5, 78.2. CFU count (mL^−1^): 900, 300, 100. Ki67 expression decreased by 20%, 35%, and 55%, respectively.	HCT116	Albino Wistar rats.	Ki67: IMT 20%, IMT-CS-NPs 35%, IMT-CS-AgNUs 55%; Zone of inhibition (*P. mirabilis*): 8.6, 12.6, 17.7 mm; Bacterial reduction at 72 h (%): *F. nucleatum* 29/44/76, *E. coli* 26/39/70, *E. faecalis* 22/37/60. Pharmacokinetics: high C_max_, AUC, t_1_/_2_ for IMT-CS-AgNUs; HET-CAM: vascular density reduced 25%, 45%, 70% (IMT, NPs, AgNUs).	[51]
CTB-PS-CS-NPs	CTB	Controlled release after 20 h; cumulative at 100 h: 100.23% ± 6.54 in PBS (pH 7.4); diffusion/erosion, reduced burst.	IC_50_: 24 h 10.10 ± 0.22 µg/mL; 48 h 5.20 ± 0.71 µg/mL.	HT-29	Balb/c mice, DMH-induced CRC; IP 10 mg/kg.	Tumor size was reduced by 71.62% at 21 days; histology showed improvement; CD31 and VEGF expression decreased; IL-6, TNF-α, and VEGF levels were lowered; mild weight loss was observed.	[52]
ALG-CS-coated MPs	CUR	Minimal release in SGF; burst in SIF due to CS dissolution; remaining payload fully released within first 2 h in SCF; overall more prolonged release vs. macroparticles.	Empty carriers are non-cytotoxic (≥80% viability); CUR-loaded significantly reduces LoVo viability, strongest at 5 mg/mL.	LoVo (human colon adenocarcinoma).	-	-	[53]
ALG-CS-coated macroparticles	CUR	<2% release in SGF; strong release in SIF; rapid complete release in SCF during early phase.	Empty carriers are non-cytotoxic; less cytotoxic than uncoated and GEL-coated macroparticles; weaker effect than CS-MPs.	LoVo (human colon adenocarcinoma).	-	-	[53]
MTX-PA-DCS NPs	MTX	pH 7.4 PBS; initial burst 7%; slower release vs. free drug (MRT: free 0.8 h, NPs 1.1 h at 5 h); kinetics fit Korsmeyer–Peppas (non-Fickian/anomalous; diffusion + polymer relaxation).	Empty NPs non-cytotoxic; MTX-PA–DCS NPs > free MTX (marked viability reduction across 10–500 μg/mL).	HT-29	-	-	[54]
Oxa-R-NGs	Oxa	US/pH-triggered controlled release; negative-to-positive charge reversal enhances tumor targeting (−9.5 to +10.4 mV from pH 7.4 to 6.5); minimal release at pH 7.4 (21.5%/24 h); faster release in acidic TME pH 6.5 (43.4%/24 h); LIU-induced ROS accelerates cargo liberation to 77.2%/24 h, confirming ultrasound-responsive disassembly.	IC_50_ = 6.5 μg/mL (CT26^luc^); Oxa-R-NGs inhibit more than free Oxa (57.0% vs. 46.5%); LIU + Oxa-R-NGs: 69.8% inhibition, 32.7% apoptosis; promotes M2 to M1 polarization (increased M1/M2 ratio).	CT26^luc^ RAW264.7	Nude BALB/c CT26 xenograft.	LIU + Oxa-R-NGs resulted in the slowest tumor growth (final volume: 828 ± 11 mm^3^ vs. controls), increased TUNEL-positive apoptosis, decreased CD31 expression, and elevated CRT and HMGB1 levels; no systemic toxicity was observed.	[55]
EV-β-CD-HA-CS-AuNCs	EV	pH-dependent release (24 h): PBS at pH 7.4, 6.5, 4.5. Sustained release with no burst. Higher cumulative release at pH 6.5 vs. 7.4 (*p* < 0.05) and highest at pH 4.5 (*p* < 0.05).	EV-β-CD-HA-CS-AuNCs: IC_50_ 16 ± 1 vs. free EV 25 ± 3 µg/mL; slight carrier cytotoxicity; rapid uptake (1–2 h); more potent migration inhibition than EV (RTCA/scratch); non-toxic to MRC-5 (free EV dose-dependent toxicity).	Caco-2 MRC-5	-	-	[56]
CL-NBSCS	CL	40% (pH 1.2), 79% (pH 5.5), 85% (pH 6.8), 75% (pH 7.4); higher release at tumor/intestinal pH.	HT-29: IC_50_ = 3.4 ± 0.82 µg/mL vs. free CL: 10.6 ± 1.14 µg/mL; L929: IC_50_ = 24.3 ± 2.23 µg/mL; enhanced cellular uptake, increased early apoptosis, and G2/M cell cycle arrest observed.	HT-29 L929	-	-	[57]

**Table 2:** Summary of ligand-modified chitosan-based delivery systems for targeted colorectal cancer therapy.

DDS	Ligand/ Receptor	Drug	Drug Release Study	In Vitro Studies		In Vivo Studies		Ref.
				Cytotoxicity	Cell Line	Animal Model	Effects	
FA-CS-5-FU NPs	FA/FR	5-FU	Sequential two-stage release test (first 2 h in pH 1.2 followed by up to 24 h in pH 6.5): FA-CS-5-FU-NPs released 17.02 ± 0.12% at 2 h (pH 1.2), and then 39.37 ± 3.00% at 2 h in pH 6.5, with an accumulative release of 96.57 ± 7.00% at 24 h; CS-5-FU-NPs released 14.50 ± 0.41% at 2 h (pH 1.2), and then 36.00 ± 2.45% at 2 h in pH 6.5, reaching 91.44 ± 7.45% at 24 h (*p* > 0.05).	IC_50_ of free 5-FU was 4.21 µg/mL, which was reduced to 3.43 µg/mL for CS-5-FU-NPs and 2.67 µg/mL for FA-CS-5FU-NPs.	Caco-2	-	-	[67]
MCSFAL224 (Zn-NMOF@CS-FA loaded with LNA-anti-miR-224)	FA/FR	LNA-anti-miR-224	50% of LNA-anti-miR-224 released within first 6 h, indicating significant early-phase release.	MCSFAL224 reduced viability to 14.2% (72 h), induced sub-G1 arrest (19.5%) and late apoptosis (67.7%), with strong upregulation of BECLIN1 (34×), BAX (36×), mTORC1 (10×), and Caspase-9 (9×), indicating potent apoptotic and autophagic response.	HCT116 CRL1831	-	-	[68]
CS-RSV-FER-FA-SLNs	FA/FR	RSV+ FER	42.87 ± 3.97% (RSV) and 45.24 ± 4.17% (FER) after 48 h (pH 7.4) biphasic drug release: initial burst (surface drug desorption and diffusion) followed by sustained release from lipid matrix.	CS-RSV-FER-FA-SLNs showed an IC_50_ of 10 µg/mL (vs. 25 µg/mL for RSV-FER-SLNs); enhanced folate receptor-mediated uptake; induced apoptosis (AO/EB staining), mitochondrial depolarization (Rh-123, DAPI), and G0/G1 arrest (68.6%). Downregulation of Cyclin D1/E and CDK2/4/6; upregulation of Bax, p53, cytochrome C, caspase-3/9, indicating intrinsic apoptosis activation.	HT-29 NIH 3T3	-	-	[69]
HA-PLGA-API-NPs	HA/ CD44	API	The uncoated PLGA-API-NPs released over 70% of the API within 24 h; HA-PLGA-API-NPs required 48 h to release 68% of the API (pH 7.4).	HRT-18 (low CD44): >80% viability up to 100 μg/mL API. HT-29 (high CD44): viability decreased to 62.36% at 10 μg/mL; cellular uptake was significantly higher in HT-29 than in HRT-18 cells.	HT-29 HRT-18	BALB/c nude (HT-29/HRT-18 xenograft)	Intravenous injection of DiR-labeled NPs: enhanced tumor accumulation vs. non-HA NPs; peak fluorescence at 8 h; minimal off-target distribution (weak heart/kidney signal); efficient active targeting and reduced systemic exposure.	[70]
5-FU@MSNPs-NH_2_/GCS	Gal/ ASGPR	5-FU	5-FU@MSNPs-NH_2_, approximately 80% and 98% of 5-FU were released in pH 7.4 after 0.5 h and 1.5 h, respectively, Fickian diffusion (n ≈ 0.25, Korsmeyer–Peppas model); 5-FU@MSNPs-NH_2_/GCS: slower release, reduced fit to first-order kinetics.	Enhanced uptake in galectin-positive SW620 cells (83.2% uptake) via Gal-receptor recognition; GC capping improves targeting specificity; 5-FU@MSNPs-NH_2_/GCS shows higher cytotoxicity (72.5% inhibition at 10 mg/mL) vs. 5-FU@MSNPs-NH_2_ (56%) and free 5-FU; induces late apoptosis (24.4%) and necrosis (14.0%); mitochondrial depolarization confirmed (JC-1 staining).	SW620	-	-	[71]

**Table 3:** Summary of main ligands used in CS-based DDS for CRC targeting.

Ligand	Receptor	Expresion	Mechanism of Action	Effect
Folic Acid	FR-α	Highly expressed in CRC; low in normal colon tissue	Binds with nanomolar affinity; triggering clathrin-mediated endocytosis	Improve uptake of drugs with poor permeability (e.g., 5-FU, IMT, polyphenols) improve uptake into FRα+ CRC cells, reduced off-target exposure; reduced systemic toxicity.
Hyaluronic Acid	CD44	Overexpressed in CRC stem-like and metastatic cells.	Binds to CD44, receptor clustering, caveolae-/clathrin-mediated endocytosis.	Enhance tumor accumulation and retention; reduced cell migration and invasion; selective cytotoxicity in CD44^+^ CRC cells.
Galactose	ASGPR	Upregulated in CRC vs. normal mucosa	Recognizes Gal motifs on nanocarrier surface; receptor-mediated uptake.	Targeted internalization into ASGPR^+^ cells; selective cytotoxicity; reduced impact on healthy fibroblasts.

**Table 4:** Comparative performance of non-targeted and ligand-modified CS-based DDS for CRC therapy.

Feature	CS-Based DDSs	Ligand-Modified CS-Based DDSs
Release kinetics	Sustained, pH-dependent release	Dual-phase release; receptor-responsive or enzymatically modulated
Cytotoxicity	Moderate	Significantly higher
Cellular uptake	Passive diffusion, electrostatic interaction, or EPR effect	Receptor-mediated endocytosis (FRα, CD44, ASGPR)
TME penetration	Limited reporting; diffusion-driven; affected by mucosal barriers	Enhanced penetration via receptor interaction and mucoadhesion
Selectivity and Toxicity	Good; mild off-target cytotoxicity; good biocompatibility	Very high selectivity for receptor-positive CRC cells; minimal fibroblast toxicity
In vivo validation	Confirmed effectiveness in many studies	Limited in vivo validation

## Data Availability

No new data were created or analyzed in this study. Data sharing is not applicable to this article.

## Supplementary Materials

The following supporting information can be downloaded at: https://www.mdpi.com/article/10.3390/md23120467/s1, Table S1: Boolean search expressions used for the literature search (January 2020–June 2025).

## Abbreviations

The following abbreviations are used in this manuscript:

5-FU	5-Fluorouracil
AgNUs	Silver Nanourchins
ALG	Alginate
AO/EB	Acridine Orange/Ethidium Bromide
API	Apigenin
ASGPR	Asialoglycoprotein Receptor
AUC	Area Under The Curve
AuNCs	Gold Nanoclusters
BVC	Bevacizumab
CD31	Cluster of Differentiation 31
CD44	Cluster of Differentiation 44
CFU	Colony Forming Unit
CL	Cyqualone
Cl	Clearance
COL	Colchicine
CPT	Camptothecin
CPT-11	Irinotecan
CRC	Colorectal Cancer
CRT	Calreticulin
CS	Chitosan
CTB	Capecitabine
CTX	Cetuximab
CUR	Curcumin
DAPI	4′,6-Diamidino-2-phenylindole
DCQAs	Dicaffeoylquinic Acids
DCS	Deacetylated Chitosan
DDSs	Drug Delivery Systems
DiR	1,1′-Dioctadecyl-3,3,3′,3′-tetramethylindotricarbocyanine iodide
DMH	1,2-Dimethylhydrazine
ENC	Encorafenib
EPR	Enhanced Permeability and Retention
ES100	Eudragit S100
ETP	Etoposide
EV	Everolimus
FA	Folic Acid
FER	Ferulic Acid
FRα	Folate Receptor-α
FTD/TPI	Trifluridine/Tipiracil
Gal	Galactose
GCS	Galactosylated Chitosan
GEL	Gelatin
GI	Gastrointestinal
GP	Genipin
HA	Hyaluronic Acid
HCT-116	Human Colorectal Carcinoma Cell Line
HCTO	Human Colon Tumor Organoids
HET CAM	Hen’s Egg Test On Chorioallantoic Membrane
HMGB1	High-Mobility Group Protein B1
HMW-HA	High Molecular Weight Hyaluronic Acid
HT-29	Human Colorectal Adenocarcinoma
HUVEC	Human Umbilical Vein Endothelial
ICG	Indocyanine Green
IMT	Imatinib Mesylate
IP	Intraperitoneal
Kp	Partition Coefficient
L100	Eudragit L100
LF	Lactoferrin
LIU	Low-intensity Ultrasound
LMW-HA	Low Molecular Weight Hyaluronic Acid
LNA	Locked Nucleic Acid
M	*miRNA*
MCTS	MultiCellular Tumor Spheroid
MFI	Mean Fluorescence Intensity
MLT	Melatonin
MMP	Mitochondrial Membrane Potential
MPECs	Magnetic Polyelectrolite complexes
MPs	Microparticles
MRT	Mean Residence Time
MSNPs	Mesoporous Silica Nanoparticles
MTX	Methotrexate
NaCMC	Sodium Carboxymethylcellulose
NBSCS	*N*-benzyl-*N*,*O*-succinyl Chitosan
NCs	Nanoclusters
NIR	Near-infrared Radiation
NIVO	Nivolumab
NPs	Nanoparticles
OA	Oleic Acid
Oxa	Oxaliplatin
P	Pectin
PA	Phytic Acid
PBMCs	Peripheral Blood Mononuclear Cells
PECs	Polyelectrolite complexes
PEG	Polyethylene Glycol
PEM	Pembrolizumab
PGA	Polyglutamic Acid
PLGA	Poly(lactide-*co*-glycolide)
PLGA	Poly(lactic-co-glycolic acid)
PNPs	Polymeric Nanoparticles
PS	Potato Starch
PTE	Pteridine Ring
RES	Reticuloendothelial System
R-NG	Charge Reversible Nanogel
ROS	Reactive Oxygen Species
RS	Retrograded Starch
RSV	Resveratrol
RTCA	Real-Time Cell Analysis
SCF	Simulated Colonic Fluid
SGF	Simulated Gastric Fluid
SIF	Simulated Intestinal Fluid
SLNs	Solid Lipid Nanoparticles
SMV	Simvastatin
t_1/2_	Half-life
TCS	Thiolated Chitosan
TME	Tumor Microenvironment
TNF-α	Tumor Necrosis Factor alpha
TPGS	D-α-Tocopheryl Polyethylene Glycol 1000 Succinate
TUNEL	Terminal Deoxynucleotidyl Transferase dUTP Nick End Labeling
US	Ultrasound
Vd	Volume Distribution
VEGF	Vascular Endothelial Growth Factor
Zn-NMOF	Zinc-based Nanoscale Metal–Organic Framework
β-CD	Beta-Cyclodextrin
