# Supplementary material for: Chitosan-Based Drug Delivery Systems for Targeted Chemotherapy in Colorectal Cancer: A Scoping Review
Source: Mar Drugs. 2025 Dec 6;23(12):467. doi: 10.3390/md23120467 (PMC12734999; doi:10.3390/md23120467)
Supplement: Supplementary file 1 [file marinedrugs-23-00467-s001.zip › marinedrugs-4010934-supplementary.pdf]

# Chitosan-Based Drug Delivery Systems for Targeted Chemotherapy in Colorectal Cancer: A Scoping Review

**Urszula Piotrowska [1,2,*], Joanna Szatko [3], Aleksandra Nowakowska [3], Emilia Klimaszewska [4], Marta Ogorzałek [4] and Marcin Sobczak [1]**

1 Department of Pharmaceutical Chemistry and Biomaterials, Faculty of Pharmacy, Medical University of Warsaw, Warsaw, 1 Banacha Str., 02-097 Warsaw, Poland
2 Department of Basic Medical Sciences, Faculty of Medical Sciences and Health Sciences, Casimir Pulaski University of Radom, 27 Chrobrego Str., 26-600 Radom, Poland
3 Students Scientific Group BIOMAT, Faculty of Pharmacy, Medical University of Warsaw, Banacha 1 Str., 02-091 Warsaw, Poland
4 Department of Cosmetology, Faculty of Medical Sciences and Health Sciences, Casimir Pulaski University of Radom, 27 Chrobrego Str., 26-600 Radom, Poland
* Correspondence: urszula.piotrowska@wum.edu.pl

**Table S1.** Boolean search expressions used for the literature search (January 2020 – June 2025).

| Section | Search string (Boolean expression) |
|---|---|
| 1. Chitosan-based drug delivery systems for colorectal cancer | "Chitosan"[MeSH Terms] AND ("Drug Carriers"[MeSH Terms] OR "Microspheres"[MeSH Terms] OR "Nanoparticles"[MeSH Terms]) AND "Drug Delivery Systems"[MeSH Terms] AND ("Antineoplastic Agents"[MeSH Terms] OR "Antineoplastic Protocols"[MeSH Terms]) AND ("Colorectal Neoplasms"[MeSH Terms] OR "Colon Neoplasms"[MeSH Terms]) |
| 2. Ligand-modified chitosan-based drug delivery systems for colorectal cancer | (chitosan[Title/Abstract]) AND<br>("drug delivery"[Title/Abstract] OR "drug delivery systems"[Title/Abstract]) AND<br>(targeting ligands[Title/Abstract] OR active targeting[Title/Abstract] OR "targeted delivery"[Title/Abstract]) AND<br>(colorectal cancer[Title/Abstract] OR colon cancer[Title/Abstract] OR colorectal neoplasm[Title/Abstract]) AND<br>(folic acid[Title/Abstract] OR hyaluronic acid[Title/Abstract] OR antibodies[Title/Abstract] OR peptides[Title/Abstract] OR aptamers[Title/Abstract] OR lectins[Title/Abstract] OR galectin[Title/Abstract] OR CD44[Title/Abstract] OR integrin[Title/Abstract] OR transferrin[Title/Abstract]) |
